# Advancements in materials, manufacturing, propulsion and localization: propelling soft robotics for medical applications

**DOI:** 10.3389/fbioe.2023.1327441

**Published:** 2024-01-08

**Authors:** Yunwen Bo, Haochen Wang, Hui Niu, Xinyang He, Quhao Xue, Zexi Li, Hao Yang, Fuzhou Niu

**Affiliations:** ^1^ School of Mechanical Engineering, Suzhou University of Science and Technology, Suzhou, China; ^2^ Department of Pathology, Second Affiliated Hospital of Soochow University, Suzhou, China; ^3^ Robotics and Microsystems Center, School of Mechanical and Electrical Engineering, Soochow University, Suzhou, China

**Keywords:** soft robotics, medical applications, intelligent materials, manufacturing methods, localization technology

## Abstract

Soft robotics is an emerging field showing immense potential for biomedical applications. This review summarizes recent advancements in soft robotics for *in vitro* and *in vivo* medical contexts. Their inherent flexibility, adaptability, and biocompatibility enable diverse capabilities from surgical assistance to minimally invasive diagnosis and therapy. Intelligent stimuli-responsive materials and bioinspired designs are enhancing functionality while improving biocompatibility. Additive manufacturing techniques facilitate rapid prototyping and customization. Untethered chemical, biological, and wireless propulsion methods are overcoming previous constraints to access new sites. Meanwhile, advances in tracking modalities like computed tomography, fluorescence and ultrasound imaging enable precision localization and control enable *in vivo* applications. While still maturing, soft robotics promises more intelligent, less invasive technologies to improve patient care. Continuing research into biocompatibility, power supplies, biomimetics, and seamless localization will help translate soft robots into widespread clinical practice.

## 1 Introduction

The definition of soft robotics has been continuously evolving since the first prototypes were developed in the late 1990s, as relevant materials, actuation technologies, fabrication procedures, and applications have adapted to new scientific advances (Suzumori et al., 1992; Tondu and Lopez, 2000). One of the most important yet simplest definitions uses Young’s modulus, ranging from 10^4^ to 10^9^ Pascals, to define “Soft” (Majidi, 2014; Rus and Tolley, 2015; Hartmann et al., 2021). This definition is well-suited to characterize the majority of early bio-inspired soft robot samples, but excludes many later developed robots that incorporate embedded rigid components. An additional advancing definition is that if a robot is constructed from materials that are relatively soft and safe compared to its operating environment, it can be classified as a soft robot, even if it is of some rigid components or structures (Cheney et al., 2015; Chen et al., 2017). To date, numerous soft robotics studies have incorporated rigid components within their actuation modules or internal structures with variable stiffness, enabling adaptation to surroundings and safe interaction with organisms (Lee et al., 2017; Medina-Sánchez et al., 2018; Thalman and Artemiadis, 2020). Potential applications for soft robots span industrial to medical scenarios, including but not limited to grasping, targeted drug delivery, monitoring, rehabilitation, function verification, diagnosis, and treatment (Rao et al., 2015; Al-Fahaam et al., 2016; Li et al., 2016a; Chen X.-Z. et al., 2017; Yang G.-Z. et al., 2019; Kim H. et al., 2020; Terzopoulou et al., 2020; Dupont et al., 2021; Zhou and Alici, 2022; Kortman et al., 2023). In particular, the possibility of using soft robots in minimally invasive therapies may represent a paradigm shift in medical treatments. Their high accessibility, adaptability, and safety when operating in the complex *in vivo* environment with multiphase physics offers significant benefits (Chautems et al., 2019; Wang C. et al., 2022).

Soft robotics has emerged as a transformative technology in the field of biomedicine, offering promising solutions for both *in vitro* and *in vivo* applications. *In vitro* (Hui and Régnier, 2011; Li et al., 2020; Zhang S. et al., 2022; Li et al., 2022), soft robots have played a pivotal role in advancing disease modeling, cell and tissue culture engineering, environmental monitoring, surgical assistance, and rehabilitation therapies. These platforms empower researchers to investigate disease processes, screen drugs, and optimize treatment plans, particularly in disease models involving the rectum, respiratory system, heart, and other vital organs. In the domain of *in vivo* applications (Li et al., 2017; Xing et al., 2020; Law et al., 2022; Ma et al., 2022; Wang et al., 2023), soft robots offer distinct advantages such as minimally invasive capabilities, compact dimensions, remote controllability, biocompatibility, and adaptability to complex fluidic environments. They hold tremendous potential in targeted drug delivery, biopsy sampling, thrombus dissolution, and minimally invasive procedures spanning various medical disciplines, including cardiology and oncology. Recent advancements have yielded innovative solutions, including magnetically actuated robotic capsules designed for site-specific drug delivery (Shimanovich et al., 2014), soft robotic capsules with fine needle biopsy capabilities for precise tissue sampling, and untethered flexible manipulators for challenging *in vivo* biopsies (Rehan et al., 2020). Soft catheters, among the most promising soft architectural designs for practical applications, are playing a pivotal role in thrombus treatment and cardiac arrhythmia intervention by facilitating direct drug delivery, cardiac tissue modulation, and even *in vivo* bioprinting. They offer minimally invasive, highly efficient solutions for addressing these critical cardiovascular conditions.

**FIGURE 1:**
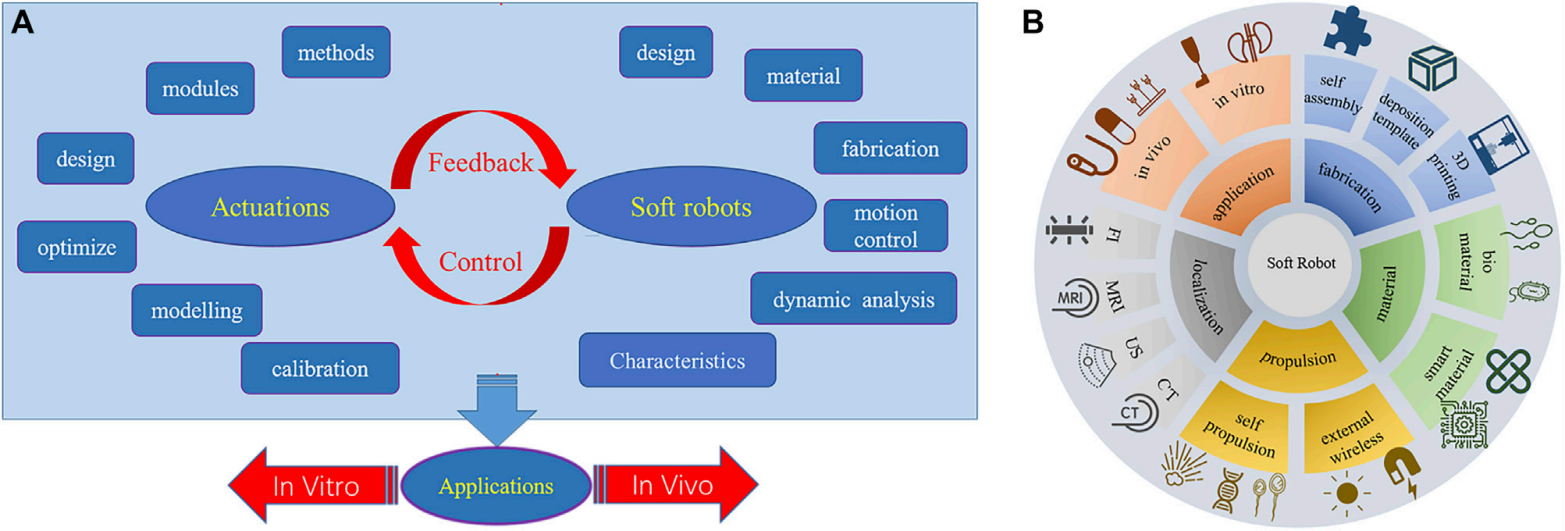
**(A)** considerations in the design and construction of soft robots. **(B)** summarized aspects in this paper.

To harness the potential applications of soft robotics, several critical factors must be taken into accounts. While soft robots can manifest in various forms, two fundamental considerations in the construction of a soft robotic system are the robot’s design and actuation, with control strategy serving as an integrative bridge between these components, illustrated in Figure 1A. The design of the robot encompasses various facets, including structural considerations, material selection, fabrication techniques, functional specifications, physicochemical characterization, kinematic properties, and biocompatibility. Materials can span a spectrum from hydrogels to elastomers, tailored to achieve desired attributes such as stiffness, haptic feedback, and biodegradability (Wang X. et al., 2022; Middelhoek et al., 2022; Chen et al., 2023). The fabrication process benefits from the use of rapid prototyping techniques like three dimensional (3D) printing and molding (Liu et al., 2015; Yang et al., 2015; Wu et al., 2016; Li J. et al., 2018; Yang et al., 2020). The second pivotal factor is actuation (Xuan et al., 2016; Gao et al., 2019; Hou et al., 2023; Tang et al., 2023), which spans options such as pneumatic systems, hydraulic systems, and smart materials like shape memory alloys (SMA) or dielectric elastomers (DE). Proper actuation involves selecting the appropriate actuation mechanism and medium, modeling and calibrating the dynamic actuation field, and precisely regulating the magnitude and distribution of forces and motions. Closed-loop feedback control connects the robot to the actuator by modulating the actuation field based on sensory inputs that monitor the robot’s state and its environment. This control mechanism facilitates the realization of the robot’s predetermined motions and functions.

Soft robotics is an emerging field that holds significant potential for medical applications due to the inherent flexibility, adaptability, and biocompatibility of soft robots compared to traditional rigid robots. This review summarizes recent advancements in soft robotics for diagnostic, therapeutic, and rehabilitative purposes, overing aspects such as applications, materials and fabrication, actuation and localization, illustrated in Figure 1B, as well as addressing challenges and prospects for the future. *In vitro* applications of soft robots outside the body include disease modeling, drug screening, surgical assistance, rehabilitation, and medical imaging. For *in vivo* applications, soft robots enable minimally invasive capabilities like targeted drug delivery, biopsy sampling, and precision surgery by navigating narrow spaces inside the body. Soft robot materials and manufacturing methods are also discussed, with a focus on intelligent materials like stimuli-responsive hydrogels and self-healing biomaterials that enhance functionality. Rapid fabrication techniques including 3D printing and self-assembly facilitate iterative design. For *in vivo* navigation systems, propulsion mechanisms can be categorized into two primary categories based on their energy sources: self-contained and external wireless propulsion. Self-contained relies on internal energy sources within the device itself, such as tendon-driven, chemical fuels, and biomechanical motion. External wireless propulsion refers to the wireless driving of the device through externally generated sources such as magnetics, acoustics, or optics. Precise localization during *in vivo* operation is enabled by tracking modalities such as fluorescence imaging (FI), magnetic resonance imaging (MRI), ultrasound (US), and computed tomography (CT) scans. Key challenges for translating soft robots into clinical practice include biocompatibility, degradability, biomimetic design, and tracking/visualization capabilities. Furthermore, with continuing research into intelligent materials, bioinspired design, propulsion, and localization, soft robots can enable the next-generation of diagnostic, therapeutic, and rehabilitative technologies.

## 2 Medical applications

Soft robots can offer several benefits over traditional rigid robots in terms of usages in medical applications. They are often more flexible and can conform to complex surfaces, and are also safer to interact with delicate tissues and organs, as they do not cause physical damage or trauma. The development of advanced soft robotics technology will likely lead to even more innovative applications in medicine. As materials science and engineering continue to advance, soft robots may become increasingly adaptable to various environments and able to perform more complex tasks. As a rapidly evolving field, robotic surgery (Li et al., 2010) not only reduces the workload for medical staffs and alleviates patients’ suffering but also finds extensive application in both *in vivo* and *in vitro* medical tasks. Soft robots exhibit substantial promise in medical applications, due to their remarkable flexibility, adaptability, and safety features. Soft robots are proficient in tasks such as precise lesion localization, minimally invasive tissue resection, targeted drug delivery, and various surgical procedures. They also hold the potential to evolve into exoskeletons for rehabilitation purposes, with a wide range of potential applications (Ding et al., 2017). This chapter will focus on summarizing the potential medical applications of soft robots.

### 2.1 In vitro

**FIGURE 2:**
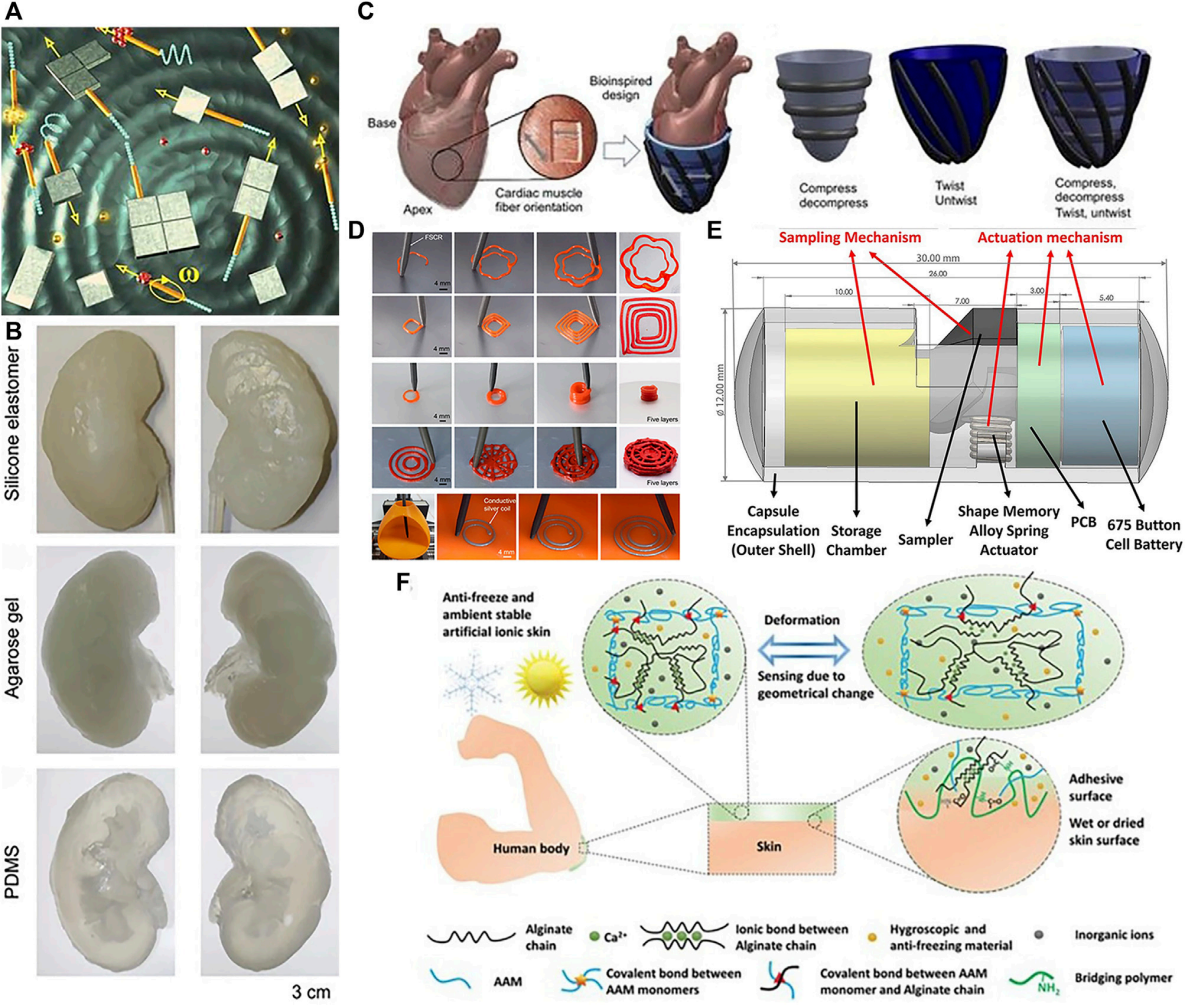
*In vitro* applications of soft robots. **(A)** Microsurgery. Reproduced with permission (Solovev et al., 2010). Copyright 2010, Wiley. **(B)** Kidney organoid. Reproduced with permission (Adams et al., 2017). Copyright 2016, ABE. **(C)** Enhance cardiac. Reproduced with permission (Roche et al., 2017). Copyright 2017, AAAS. **(D)** Ink writing. Reproduced with permission (Zhou C. et al., 2021). Copyright 2021, Springer Nature. **(E)** Capsule robots that can perform biopsy sampling. Reproduced with permission (Rehan et al., 2020). Copyright 2020, Wiley. **(F)** Artificial ionic skins. Reproduced with permission (Ying et al., 2021). Copyright 2021, Wiley.

*In vitro* medical applications of soft robots include but not limit to: environmental monitoring (Rao et al., 2015; Chen X.-Z. et al., 2017), surgical assistance (Kim H. et al., 2020), rehabilitation therapy (Al-Fahaam et al., 2016), targeted delivery (Li et al., 2022), developing disease models (Roche et al., 2017; Zrinscak et al., 2023), functional structures (Calderon et al., 2019; Pang et al., 2021; Ying et al., 2021) and tissue engineering (Zhou Y. et al., 2021). Solovev et al. (2010) wirelessly controlled microrobots using external magnets to assist with loading, transport, delivery, and assembly of microparticles and nanosheets in fuel solutions, shown in Figure 2A. Given ethical constraints, direct *in vivo* experimentation with humans can be highly complex, so establishing accurate *in vitro* disease models is crucial for furthering soft robotics in biomedicine. In Zrinscak et al. (2023), the authors introduce disease models of the gastrointestinal tract, respiratory system, cardiovascular system, and other organs amenable to soft robotics research. Adams et al. (2017) fabricated a kidney using diverse materials, illustrated in Figure 2B. Roche et al. (2017) developed a device to enhance cardiac function and performed *in vitro* experiments shown in Figure 2C. Zhou C. et al. (2021) achieved direct ink writing of flexible, stretchable conductive traces via ferromagnetic soft catheter robot (FSCR), shown in Figure 2D. Rehan et al. (2020) conceptualized a computer aided design (CAD) based capsule robot design comprised of an actuation mechanism and sampling mechanism shown in Figure 2E. With soft robotic cell culture platforms, clinicians and scientists can better study disease processes, screen pharmacological agents, and optimize therapeutic regimens. Another promising exterior application is wearable devices to provide ambulatory assistance and gait rehabilitation for patients with mobility impairments (Li et al., 2018). Compared to rigid exoskeletons, soft exoskeletons are lighter, more compliant, and less restrictive of the user’s natural movements, promising greater comfort. As one example, Yang X. et al. (2019) developed a soft, continuous exoskeleton mimicking the spine to aid with bending and lifting activities, designed to conform to human anatomy and reduce forces on the back. Ying et al. (2021) designed artificial ionic skins with multifunctional strong adhesives that exhibit high stretchability, anti-freezing ability, and environmental stability in Figure 2F. Moreover, soft robotics can enhance imaging techniques. A novel soft robotic effector was proposed aiming at safely obtain standard views required for prenatal diagnostic fetal US exams. Its adjustable shape conforms to varied maternal anatomies, enabling more flexible and reliable fatal imaging (Lindenroth et al., 2020). In summary, while soft robots demonstrate immense potential in *ex vivo* contexts, their *in vivo* applications are more anticipated. However, at present, this field is still in its infancy, experiencing both laboratory demonstrations and industrial development. It still leaves a last step for being truly utilized in medical applications. Currently, for achieving *in vivo* applications, there often lack sufficient strength, stability, and precision to perform complex surgical tasks. Additionally, their sensors and control systems require further improvement to achieve more accurate and reliable operation.

### 2.2 In vivo

**FIGURE 3:**
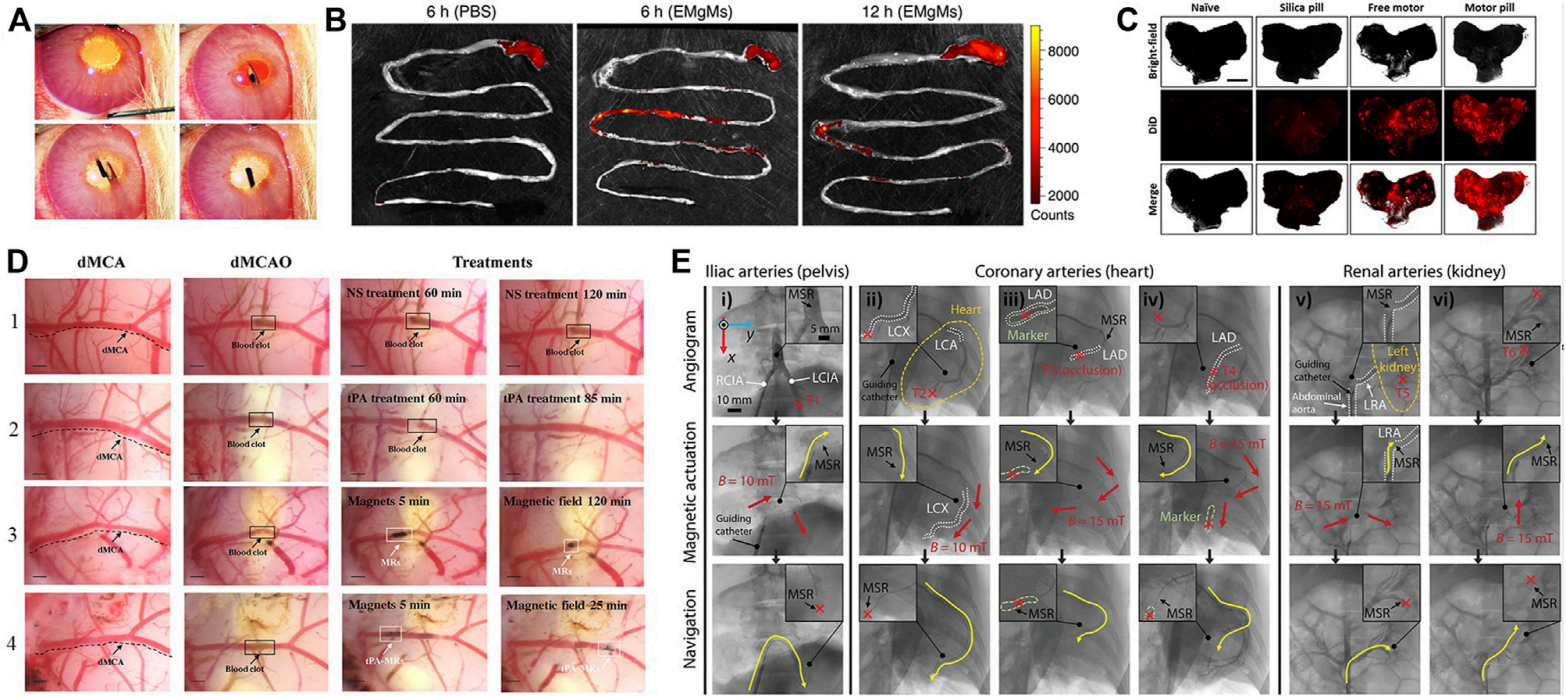
*In vivo* applications of soft robots. **(A)** Work in vitreous. Reproduced with permission (Pokki et al., 2017). Copyright 2016, Wiley. **(B)** Targeted delivery in Gastrointestinal. Reproduced with permission (Li et al., 2016b). Copyright 2016, ACS. **(C)** Targeted delivery in stomach. Reproduced with permission (Karshalev et al., 2018). Copyright 2018, ACS. **(D)** Thrombolysis by tissue plasminogen activator (tPA) microrods. Reproduced with permission (Hu et al., 2018). Copyright 2018, ACS. **(E)** Navigation of microrobotic guidewire. Reproduced with permission (Hwang et al., 2022). Copyright 2022, Wiley.

Soft robots enable unique capabilities for minimally invasive endoscopic surgeries (Gifari et al., 2019), permitting direct puncturing, cutting, or extracting of cells and tissues with extreme precision down to the cellular scale. Compared to bulky rigid robots, soft robots can traverse narrow blood vessels and anatomical tracts to access lesions at hard-to-reach interior locations. Advantages like small sizes (Ren et al., 2021), remote controllability (Zhou C. et al., 2021), biocompatibility, adaptability to low Reynolds number fluid environments (Digumarti et al., 2018), and facile conversion of external power to motion make soft robots well-suited for *in vivo* use. Consequently, soft robots hold tremendous potential for targeted drug delivery, biopsy sampling, thrombus dissolution and other minimally invasive procedures (Wang et al., 2017), while continuing to break new ground in cardiology (Chautems et al., 2019), oncology (Forbes, 2010; Luo et al., 2016) and other medical fields. Current *in vivo* soft robotic systems mainly take the forms of capsules (Basar et al., 2018), catheters, microgrippers, and micro/nanomotors that harness nanotechnology for precise motion control. Pokki et al. (2017) validated the potential of soft robots, which can be injected into rabbit, for ocular disease diagnosis, therapy, and drug delivery, shown in Figure 3A. Achieving localized targeted drug delivery (Shimanovich et al., 2014) can effectively avoid these pitfalls, spurring intense research in biomedicine. Zhou and Alici (2022) proposed a magnetically actuated robotic capsule for site-specific drug release in the gastrointestinal tract. The capsule contains an extendable needle to directly inject drugs into diseased tissues, enabling rapid absorption and enhanced delivery efficacy. Lu et al. (2018) designed soft robots capable of rapid locomotion in environments like the stomach, enabling targeted drug delivery via cargo capsules. Additionally, a multilayered microrobot termed metal-organic framework (MOF) to qualify as small-scale robot was developed, with each layer providing distinct functionality (Terzopoulou et al., 2020), it enables targeted drug delivery by loading therapeutics into biodegradable drug carriers. Preliminary depictions have emerged for future integrated application scenarios of 3D printing *in vivo* and biodegradable microrobotic swimmers (Ceylan et al., 2019). Li et al. (2016b) proposed an intestinal micromotor system using micromotors coated with an enteric polymer layer to convey payloads to specific locations. Propulsion activated at the target site enabled local tissue penetration and retention, as shown in Figure 3B, achieving directional transportation in the jejunum. Similarly, Karshalev et al. (2018) proposed micromotor pills for cargo delivery focused on the stomach, achieving higher retention compared to other methods as shown in Figure 3C.

Common tissue sampling techniques like surgery, needle aspiration, and biopsy carry procedural risks while often yielding insufficient or inaccurate samples affected by surrounding tissues. With advances in *in vivo* soft robotics, soft robots guided by imaging can access tumor or lesion sites to perform tissue and cell sampling in a safer, more efficient, and thorough manner, significantly enhancing diagnostic accuracy and utility. For example, a magnetically-driven soft robotic capsule for fine oligonucleotide analogs can capture and isolate proteins. Additionally, antibody-coated microrockets allow selective capture and separation of cancer cells for diagnosis and therapy (Li et al., 2016a). Diller and Sitti (2014) proposed untethered, precisely manipulative microgrippers capable of non-invasive access to confined spaces for executing out-of-plane 3D operations and assembly tasks to create complex 3D materials and structures.

Medical catheters can administer thrombolytic drugs or physically intervene in cardiac tissues to enable treatment of thrombosis and cardiac arrhythmias. Thrombosis, characterized by clot formation obstructing blood vessels, is a high-incidence cardiovascular disease warranting novel therapies. As a high-incidence cardiovascular disease, thrombus treatment has attracted much attention. Thrombi are solid clotted masses formed from components in the blood that block blood vessel lumens, disrupt blood flow and thus cause other cardiovascular diseases. Conventional treatments like anticoagulants, thrombolytics, aspiration, surgical removal, or stenting each have limitations in invasiveness, efficacy, or recurrence risk. Ideal thrombosis treatment should rapidly and thoroughly dissolve clots in a minimally invasive manner without increasing bleeding risk. Hu et al. (2018) overcame limitations of standalone tPA thrombolysis using modified tPA-loaded microrobots, significantly improving thrombolytic efficacy in Figure 3D. Intravenously injected microrobots could be renally excreted without damaging kidneys or liver, enabling effective treatment for ischemic stroke. Soft robotic catheters hold great potential for *in vivo* thrombolytic interventions. In Figure 3E, Hwang et al. (2022) proposed a microrobotic system for real-time remote manipulation of micro-guidewires by physicians. The multifunctional soft robot catheter put forward (Rogatinsky et al., 2023) can achieve stable, dexterous, and efficacious performance within the heart, surmounting core impediments stemming from dimensional disparities, maneuverability requirements, and remote operability, while concurrently expanding possibilities for minimally invasive intracardiac procedures. Clinical studies in coronary, iliac, and renal arteries demonstrated feasibility and effectiveness. Similarly, arrhythmias arising from irregular cardiac electrical patterns can be addressed by soft cardiac ablation devices capable of precise electrophysiology modulation. Beyond drug delivery and tissue intervention, soft robotic catheters also demonstrate promise for *in vivo* bioprinting. For example, Zhou C. et al. (2021) utilized a magnetically-driven soft catheter to conduct minimally invasive bioprinting inside the body.

## 3 Materials and manufacturing

A crucial distinction between soft robots and traditional rigid robots is the utilization of highly stretchable, flexible materials. The most common materials used in soft robotics are silicone, elastomers, and hydrogels. Silicone robots are made from a flexible and durable silicone material, while elastomer robots are made from a viscoelastic material that can be stretched and compressed. Hydrogel robots are composed of a water-based gel that can be easily molded and shaped. Ideal materials possess high elongation (>200%) and low Young’s modulus (0.1–10 MPa) (Rus and Tolley, 2015; Hartmann et al., 2021; Zhu et al., 2023), while Polydimethylsiloxane (PDMS) (Shepherd et al., 2011; Gossweiler et al., 2015; Lu et al., 2018) and Ecoflex (Shepherd et al., 2011; Rusu et al., 2023) are prevalent in existing soft robots. We highlight emerging smart materials that could enhance soft robotic performance and efficacy in this chapter. In particular, stimuli-responsive hydrogels (Panda et al., 2023) capable of altering their shape or mechanical properties on demand could enable soft robots to adapt to dynamic *in vivo* environments. We summarized the materials involved in this paper in Supplementary Table S1.

### 3.1 Materials

**FIGURE 4:**
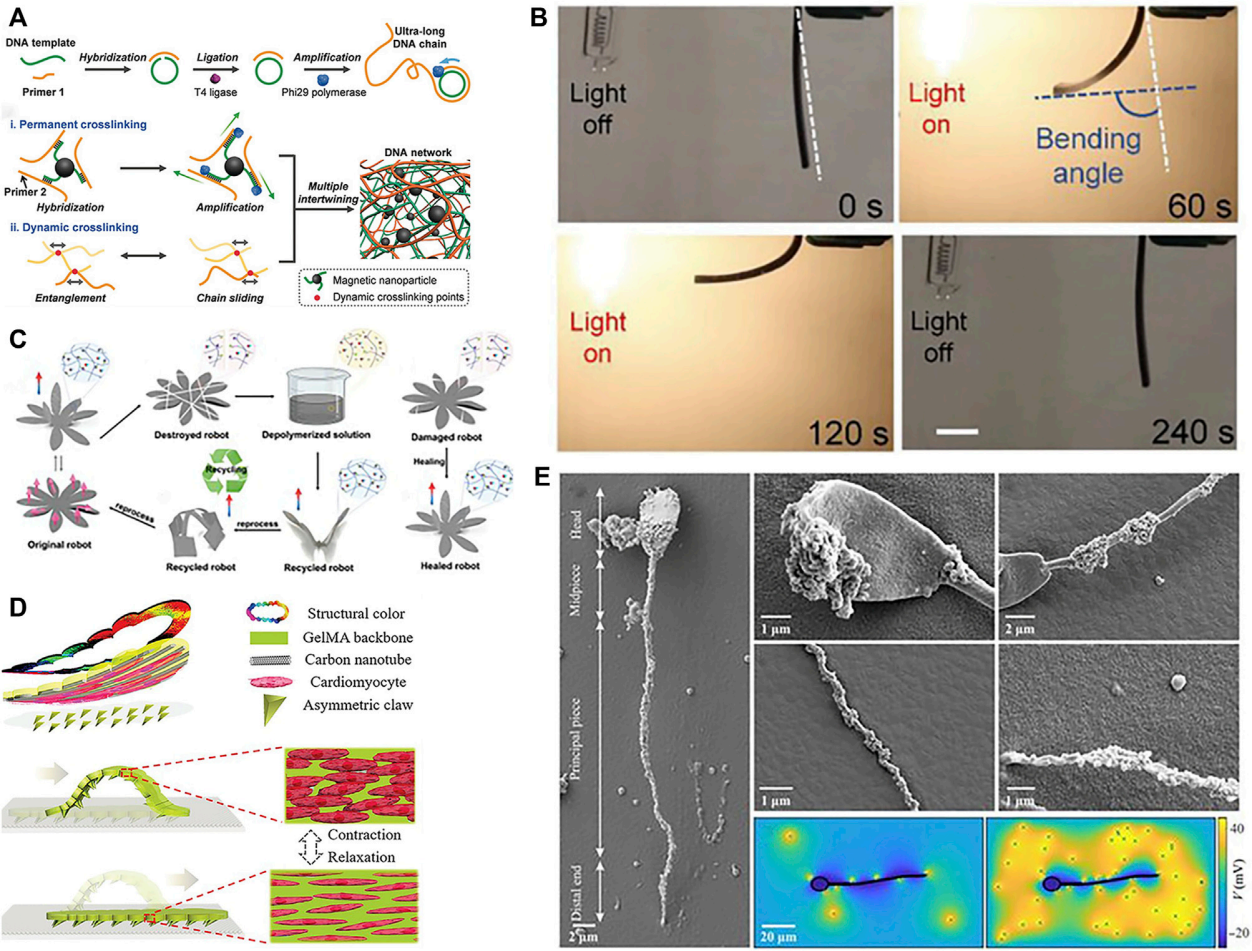
Materials of soft robots. **(A)** DNA. Reproduced with permission (Tang et al., 2020). Copyright 2019, Wiley. **(B)** Optical-stimulated material. Reproduced with permission (Ahn et al., 2019). Copyright 2019, Wiley. **(C)** Self-healing material. Reproduced with permission (Zhu et al., 2023). Copyright 2023, Wiley. **(D)** Cardiomyocytes. Reproduced with permission (Sun et al., 2019). Copyright 2019, Wiley. **(E)** Sperm. Reproduced with permission (Magdanz et al., 2020). Copyright 2020, AAAS.

Smart materials capable of sensing and responding to external stimuli are enabling new paradigms in soft robotics. As novel materials that can respond to external stimuli such as temperature, light, US, pH, ions, and magnetic fields by changing their properties or functions accordingly. Common smart materials include poly(N-isopropylacrylamide) (PNIPAM), liquid crystal elastomers (LCE), SMA (Song et al., 2016), MOF (Ikezoe et al., 2015), macromolecules (Tang et al., 2020), etc. Hydrogels are a versatile responsive material, exhibiting significant volume change under temperature, pH, light, and other triggers. Composite hydrogels confined with other materials can direct strain to targeted regions or directions, imparting multifunctional responsiveness (Jeon et al., 2017; Banerjee et al., 2018). The incorporation of conductive fillers can impart conductivity to hydrogels. Conductive nanofillers can provide resistivity-based tactile sensing and, via composite formation, stimulate actuation. Moreover, doping with other materials in the hydrogel matrix can enable actuation capabilities (Zhou et al., 2018). In order to enhance biocompatibility more effectively, efforts have been made to optimize biocompatibility, biocompatible hydrogels like gelatin methacryloyl (GelMA), derived from enzymatically degradable gelatin, represent a promising alternative to poly(ethylene glycol) diacrylate (PEGDA) for fabricating soft microrobotics. GelMA polymer is derived by functionalizing gelatin (a denatured and partially hydrolyzed polypeptide mixture) and can substitute PEGDA for fabricating helical microstructures. Gelatin can be digested by proteases like matrix metalloproteinase-2 (MMP-2). Compared to PEGDA, GelMA exhibits lower toxicity (Wang et al., 2018; Ceylan et al., 2019). MOFs are highly porous crystalline coordination polymers possessing desired traits for motile micro/nanodevices including high payload capacity, biodegradability, biocompatibility, and stimulatory responsiveness, can efficiently transduce stimuli into micro/nanorobot motion. Zhang X. et al. (2022) reported a near-infrared light-driven, shape-programmable hydrogel actuator by loading MOFs on a PDMS film, achieving distinct shape changes under near-infrared irradiation, and demonstrated how to fabricate MOF. Tang et al. (2020) introduced a deoxyribonucleic acid (DNA) nanorobot where DNA molecules can self-assemble and self-organize into stable structures through interactions. Moreover, the programmability of DNA allows specific functions via sequence synthesis and modification. This DNA hydrogel robot exhibits ultrasoftness and supertoughness, enabling shape adaptivity and magnetically-driven navigation in confined, unstructured spaces in Figure 4A. In addition, Ahn et al. (2019) fabricated liquid crystal elastomer-carbonate/carbon nanotube (LCE-CNT) composite films capable of reversible photoinduced bending shown in Figure 4B. Sun et al. (2022) demonstrated magnetically driven mucus microrobots using non-Newtonian fluids, which can achieve functions including grasping solid objects, swallowing and transporting harmful substances, monitoring human body movement, as well as circuit switching and repairing.

Further advancements in smart materials may enable additional capabilities, such as bio-inspired designs or self-healing functions. For example, Rogóż et al. (2016) harnessed the photosensitivity of LCE to create a soft, caterpillar-like robot driven by asymmetric illumination. The robot’s segmented body deforms under patterned light to inch forward through peristaltic motion. In another demonstration, Mao et al. (2014) activated flexible SMA rays in a starfish-inspired soft robot capable of multi-modal locomotion and obstacle clearance twice its height. By combining responsive materials with bioinspired designs, these robots exemplify how smart material actuation can achieve lifelike motion in soft-bodied systems. In particular, self-healing materials hold particular promise for enhancing soft robot robustness during prolonged *in vivo* operation (Terryn et al., 2021). Polyimide (PI) is a high-performance polymer renowned for its outstanding thermal stability, mechanical robustness, and chemical resistance. The Young’s modulus of PI is typically between 2.5 and 6 GPa, depending on factors like polymer structure, manufacturing technique, and temperature. When incorporated into composites, PI’s excellent mechanical properties and deformability enable its use in developing soft robots that can function for prolonged periods *in vivo* (Rubehn and Stieglitz, 2010; Dong et al., 2013). Zhu et al. (2023) introduced a novel dynamic covalent PI with softness, stretchability, recoverability, rapid room-temperature self-healing, and multimodal actuation capabilities. By reducing crosslink density and utilizing intermolecular hydrogen bonding, the polymer matrix achieved softness, stretchability, recoverability, and rapid self-healing at room temperature. Through the addition of magnetic particles, wireless magnetically-controlled actuation was realized in soft robots under external magnetic fields shown in Figure 4C.

In addition, biomaterials with good biocompatibility and degradability are also gradually being studied by scholars. Common biological materials that can be used for soft robot fabrication include algae, bacteria, sperm, cardiomyocytes (Sun et al., 2019), spores, etc. Sun et al. (2019) achieved crawling of soft robots driven by cardiomyocytes in Figure 4D. Sareh et al. (2013) mimicked the flagella of the alga Volvox to develop planar actuators. By optimizing the segmentation patterns and bioinspired driving signals, they successfully replicated the motion of natural cilia in artificial cilia. These exhibited good performance in low Reynolds number environments. Magdanz et al. (2020) introduced a hybrid magnetic microrobot using self-assembled non-motile sperm cells and magnetic nanoparticles. These function as biocompatible, controllable, and detectable biohybrid tools with potential for targeted *in vivo* therapies shown in Figure 4E. Justus et al. (2019) engineered a soft gripper containing engineered bacteria, a flexible light-emitting diode (LED) circuit, and a soft pneumatic actuator. Their study demonstrated that the bio-LED-actuator module can detect chemical signals by pressurizing and releasing contents of a hydrogel injected with chemicals. It can also make viable decisions using chemical sensing and feedback during pick-and-place operations, and integrating chemically responsive synthetic cells and soft materials for biosensing soft robots. As these cases illustrate, harnessing biological building blocks and bioinspired designs can impart unique capabilities to soft robots while ensuring biocompatibility.

### 3.2 Manufacturing methodologies

**FIGURE 5:**
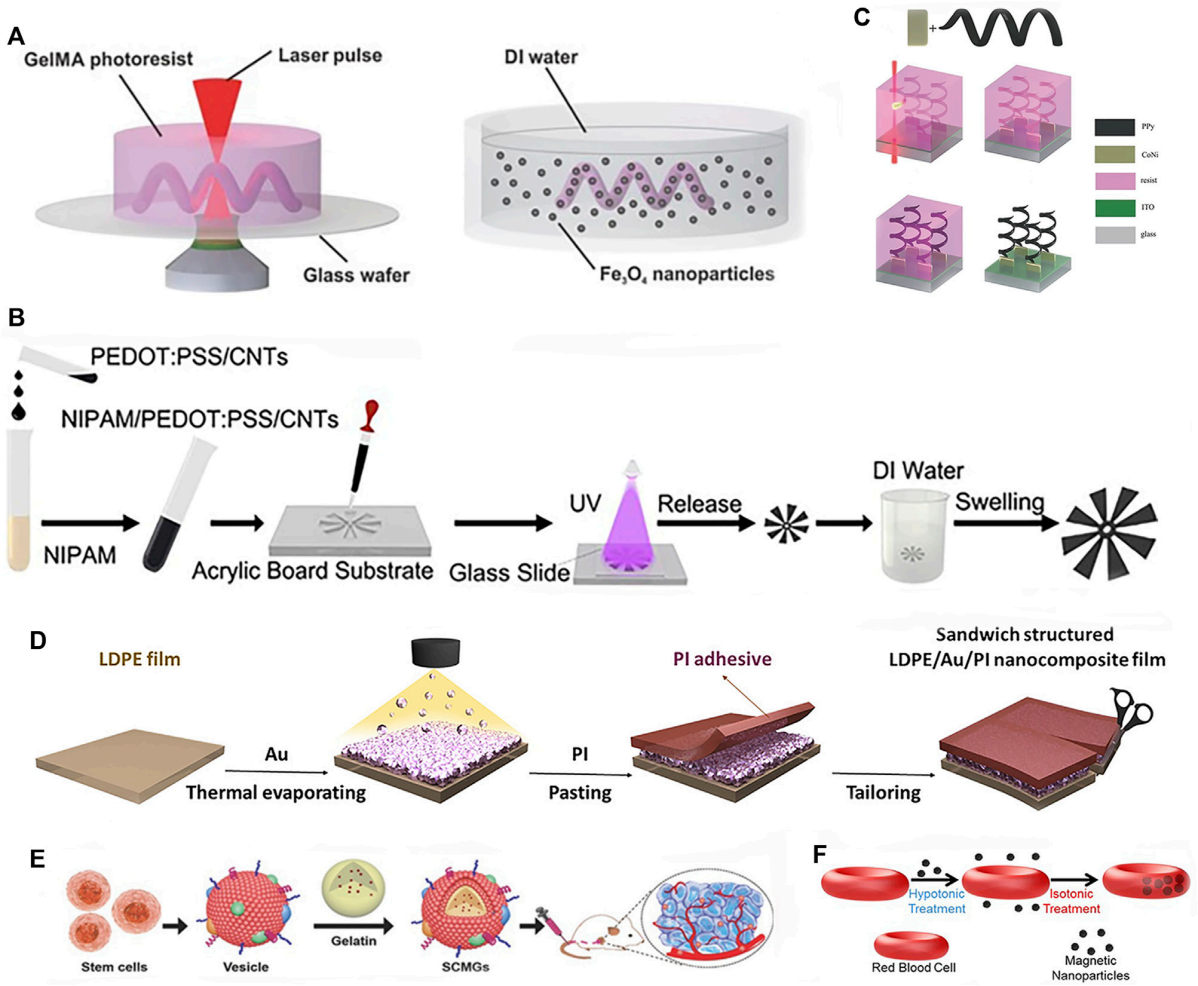
Fabrications methods of soft robots. **(A)** 2PP. Reproduced with permission (Wang et al., 2018). Copyright 2018, Wiley. **(B)** Photopolymerized and template. Reproduced with permission (Yin et al., 2021). Copyright 2021, ACS. **(C)** Template, electrodeposition and electropolymerization. Reproduced with permission (Zeeshan et al., 2014). Copyright 2013, Wiley. **(D)** Deposition. Reproduced with permission (Yu et al., 2021). Copyright 2021, Wiley. **(E)** Assembly. Reproduced with permission (Gao et al., 2016). Copyright 2016, Wiley. **(F)** Self-assembly. Reproduced with permission (Wu et al., 2014). Copyright 2014, ACS.

Rapid fabrication technologies like 3D printing (Joyee and Pan, 2019) and shape deposition modeling enable accelerated design iterations compared to conventional manufacturing. Wang et al. (2018) printed helical microstructures using two-photon polymerization (2PP). Next, incubating these microstructures in an aqueous suspension of magnetic iron oxide (Fe_3_O_4_) nanoparticles imparted magnetism, yielding biodegradable GelMA helical microrobotic swimmers shown in Figure 5A. Yin et al. (2021) fabricated visible light-driven jellyfish-like miniature soft robot (JMSR) robots using molds and ultraviolet (UV) light shown in Figure 5B. By facilitating agile modification of product categories and dimensions, these advanced techniques are well-suited for iteratively prototyping soft robots with complex geometries and functionalities. The rapid prototyping of soft robots is being facilitated by numerous techniques, including 3D printing, templated deposition, and self-assembly methods. These approaches allow for the expedited fabrication of soft robot prototypes while also creating new opportunities for customized medical devices. In the following sections, we review recent advances in these promising fabrication paradigms.

3D printing enables multi-material fabrication to achieve complex structures and geometries, while providing high degrees of freedom and design flexibility. By avoiding complex machining and assembly in traditional manufacturing methods, Zhou et al. (2022) successfully printed an intermediate skeleton for a continuous robot with rigid-soft-rigid structures. Photolithography holds great promise in the fabrication of soft robots. This advanced light-based manufacturing technique demonstrates remarkable precision, scalability, material compatibility, integration of functionalities, and design flexibility, making it particularly suitable for soft robot manufacturing (Qin et al., 2014). The recent advancements in laser lithography technology and molecular alignment engineering have enabled the arbitrary 3D pattern design of LCE. By harnessing predetermined driving characteristics, these engineered LCE structures can exhibit a diverse range of motions as a cohesive unit (Rogóż et al., 2016). In addition to the aforementioned techniques, there are other manufacturing methods. The fabrication of soft robots can also be achieved through templated deposition or assembly/self-assembly approaches. Templated deposition utilizes a sacrificial mold or template to guide the structured build-up of materials, enabling precise control over the resulting structure. Meanwhile, assembly or self-assembly methods can be used to construct soft robots by integrating and joining components either manually or automatically. Utilization of electrodeposition and electropolymerization technique in conjunction with 3D template assistance resulted in the fabrication of hybrid helical microrobots shown in Figure 5C (Zeeshan et al., 2014). Yu et al. (2021) proposed a light-responsive nanocomposite thin film that can be used for mass production of sandwich-structured devices, as shown in Figure 5D. To improve biocompatibility for better *in vivo* application, incorporating biomaterials through assembly/self-assembly approaches can effectively enhance bio-compatibility. Gao et al. (2016) developed bone marrow-derived mesenchymal stem cell membrane cloaked gelatin nanogels as an efficient tumor-targeting drug delivery platform, as shown in Figure 5E. Wu et al. (2014) fabricated red blood cell micromotors using a self-assembly approach, as depicted in Figure 5F. A novel approach is presented for the development of a bio-hybrid magnetic microrobot utilizing electrostatic self-assembly of non-motile sperm cells and magnetic nanoparticles (Magdanz et al., 2020). A method combining ordered self-assembly and sol-gel reaction was introduced to fabricate bio-inspired silica honeycomb membranes with controlled structural and chemical characteristics (Aynard et al., 2020). Overall, these advanced fabrication paradigms are overcoming traditional manufacturing limitations to unlock new horizons in soft robot design and performance.

## 4 Propulsion and localization

Appropriate soft robotic actuation and localization depend on the intended *in vivo* versus *in vitro* application. For external devices like exoskeletons, tethered fluidic or tendon-driven actuators suffice (In et al., 2015). However, for *in vivo* applications, traditional tethered soft robots are gradually being replaced by untethered propulsion methods due to invasiveness concerns. Untethered actuation can be further classified as self-contained or external wireless, depending on whether the power source is internal or external to the body. Unlike *ex vivo* soft robots, determining position and orientation is critical for *in vivo* soft robots. Current imaging modalities explored for soft robot localization include US, MRI, FI, CT scans, etc. In particular, swarm robotics with multiple miniature soft robots can enhance localization through collective imaging. In summary, choosing suitable energy sources and imaging techniques based on the intended environment will maximize functionality and biocompatibility of soft robots for practical applications ranging from drug delivery to minimally invasive surgery.

### 4.1 Propulsion methodologies

**FIGURE 6:**
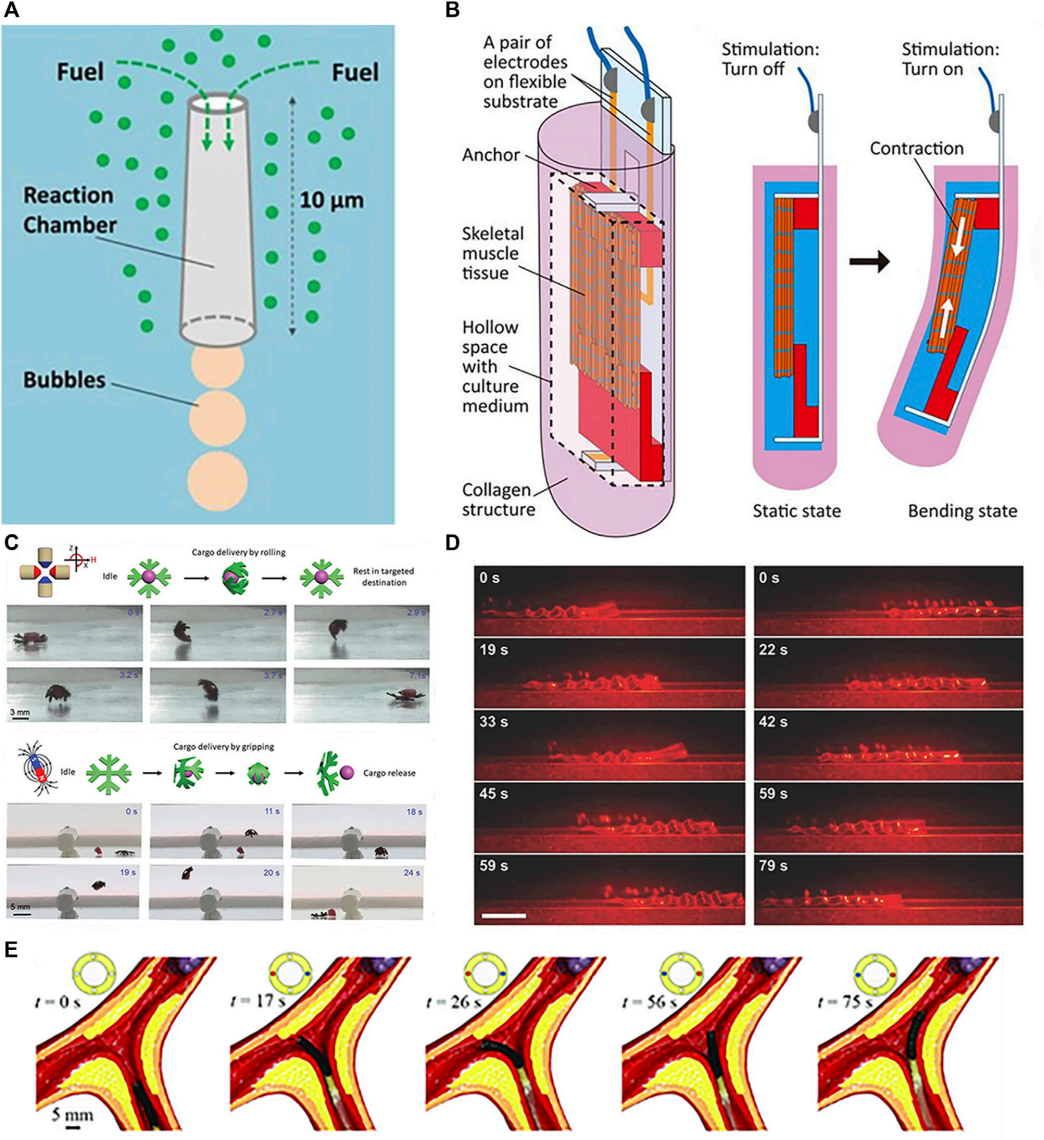
Propulsions of soft robots. **(A)** Propulsion by chemical. Reproduced with permission (Li et al., 2016a). Copyright 2016, ACS. **(B)** Propulsion by muscle tissue. Reproduced with permission (Morimoto et al., 2020). Copyright 2020, APL. **(C)** Propulsion by magnetic. Reproduced with permission (Goudu et al., 2020). Copyright 2020, Wiley. **(D)** Propulsion by optical. Reproduced with permission (Rogóż et al., 2016). Copyright 2016, Wiley. **(E)** Propulsion by tendon. Reproduced with permission (Zhang et al., 2021). Copyright 2020, Wiley.

Actuating soft robots externally using fluids and tendons has matured considerably. Roche et al. (2017) utilized compressed air to actuate silicone artificial muscles for compression and twisting, mimicking normal cardiac motions to assist failing hearts. Phan et al. (2022) presented hydraulically actuated artificial muscle fibers for smart textiles with high plasticity, adaptivity, and mechanical programmability for multimodal motion and shape shifting. The focus now shifts towards internal propulsion mechanisms. Self-contained is the ability to produce on-board thrust for autonomous motion. Initially studied for microparticles via chemical reactions or external field excitation, self-contained has recently been applied in biomedicine with nanotechnology and microrobotic advancements. For soft robots, internal propulsion can be categorized into chemically-driven, biologically-driven self-contained or wireless propulsion. Chemical driving involves asymmetric bubble release from reactions, self-electrophoresis, or diffusiophoresis from local concentration and potential gradients at the surface (Moran and Posner, 2017). Comprised of a chemical catalyst surface and a narrow channel holding chemical fuel, reactions occur as fuel flows over the catalyst, generating bubbles that asymmetrically emit from a wider outlet to produce thrust. By tuning conditions like the reaction and channel geometry, microrocket motion can be controlled in Figure 6A (Li et al., 2016a).

Biological driving integrates biological and non-biological materials, exploiting innate or environmental taxis for propulsion. Commonly used bio-components include sperm, bacteria, algae, and cardiomyocytes. Singh et al. (2020) developed a sperm-propelled microrobot that harnesses flagellar beating for thrust with inherent biocompatibility and high speeds, overcoming propulsion and compatibility challenges. Bacterial motility is highly efficient and controllable at the microscale, enabling autonomous navigation in complex fluids (Carlsen and Sitti, 2014). Exploiting bacterial taxis and extracellular responses enables precise drug release in tumor microenvironments for cancers like hepatocellular carcinoma, colorectal cancer, and prostate cancer (Forbes, 2010). Careful concentration control prevents toxic immune reactions (Hosseinidoust et al., 2016). Zhuang et al. (2015) utilized bacterial pH taxis for propulsion. Like bacteria, algae exhibit taxis. Weibel et al. (2005) attached payloads (1–6 μm polystyrene microparticles) to phototactic Chlamydomonas reinhardtii and steered the loaded swimming cells via phototaxis for light-triggered cargo release. Sun et al. (2019) fabricated a cardiomyocyte-powered robot where cardiomyocytes interacted with carbon nanotube layers to generate directional motion via asymmetric leg designs. Shown in Figure 6B, Morimoto et al. (2020) encapsulated skeletal muscle tissue in collagen structures, achieving locomotion through contraction of the muscle tissue in the collagen. Skeletal muscle actuation is also possible (Morimoto and Takeuchi, 2022).

External wireless propulsion is an active research area focusing on magnetic (Frutiger et al., 2009; You et al., 2021), optical (Ahn et al., 2019; Yin et al., 2021), and acoustic (Xu et al., 2017; Zhou Y. et al., 2021) actuation. Magnetic driving generates forces or torques using magnetic field gradients or alternating fields, which can modulate velocity and trajectories through changes in field strength and direction. This enables motions like rolling, tumbling, precession, corkscrewing, and travelling-wave propulsion (Nelson et al., 2010). Hua et al. (2022) proposed a novel magnetically actuated FSCR for intragastric diagnosis and therapy. The FSCR uses a composite shell of ferrofluid, permanent magnets, and soft elastomers remotely actuated by external permanent magnets, the key features include: 1) The composite shell has a soft outer surface to reduce tissue damage while improving magnetic circuits for controlled, safe motion via the ferrofluid-magnet combination. 2) The FSCR integrates an oscillation module for advanced functions like drug release. Acoustic waves oscillate microswimmers via applied ultrasonic forces. Goudu et al. (2020) demonstrated magnetic actuation of untethered hydrogel microrobots for grasp, transport, and release of cargo shown in Figure 6C. Ultrasound propulsion offers excellent controllability and tunability by modulating parameters like frequency, amplitude, and duration to precisely control speed and direction (Rao et al., 2015). Real-time control, longevity, non-invasiveness, wireless, and biocompatibility enable precise drug delivery and imaging via micro/nanorobotic manipulation, including ultrasonic excitation and oscillations in acoustic suspensions (Xu et al., 2017). Terzopoulou et al. (2020) alternately activated/deactivated UV-vis light to produce walking motions of a ribbon-like MOF film through cyclic swelling and shrinking. Rogóż et al. (2016) fabricated LCE soft robots capable of photo-driven actuation shown in Figure 6D. Light can also generate secondary effects like photothermal responses (Terzopoulou et al., 2020).

In addition to individual approaches, hybrid actuation combines multiple integrated mechanisms for enhanced versatility (Kim D.-I. et al., 2020; Hu et al., 2022). Zhang et al. (2021) demonstrated active steering of tendon-magnetically driven soft continuum robots through tendon actuation in a vascular model shown in Figure 6E. For example, Laschi et al. (2012) coupled tendons with SMA to achieve both longitudinal and lateral driving in a robotic arm. Yuan et al. (2020) created Janus micromotors with two dimensional (2D) nanomaterial coatings responsive to chemical fuels, light, and magnetism. Selective coating of the hemispherical motors enabled a bubble propulsion engine powered by catalytic nanoparticles, a magnetic engine propelled by iron oxides, and an optical engine driven by quantum dot illumination. Such multifunctional hybrid systems overcome limitations of individual actuation modes for more robust soft robotic control.

### 4.2 Localization and tracking

Unlike *ex vivo* settings, determining soft robot position and orientation is critical for *in vivo* targeting and navigation. Currently pursued medical imaging modalities for soft robot localization include FI (Luo et al., 2016; Singh et al., 2017), MRI (Böll et al., 2019), US (Wang and Zhang, 2020), and CT (Masamune et al., 2001). Each approach has tradeoffs in factors like resolution, field-of-view, accessibility, and compatibility with soft materials that guide appropriate matching to the clinical application. For instance, while MRI provides excellent soft tissue contrast, the magnetic fields may interfere with soft robots incorporating ferromagnetic components.

FI provides high detection sensitivity for tracking soft robots labeled with appropriate fluorescent markers. It allows real-time monitoring of dynamic changes in target substances (Singh et al., 2017) and facilitates the acquisition of multi-channel image information by simultaneously utilizing multiple fluorescence dyes with different excitation wavelengths and emission filters. However, the presence of intrinsic fluorescence can interfere with the fluorescence signal of the target substance. Similarly, the imaging capability in deep tissues is constrained by attenuation and scattering, and heavily relies on the efficiency and specificity of fluorescence dye selection and labeling. Servant et al. (2015) monitored magnetic navigation of microswimmers using near-infrared probes (NIR-797) dye-labeled artificial bacterial flagella (ABFs). FI is a versatile localization modality, but optimal dye selection and tissue depth constraints warrant consideration.

MRI is a technique that utilizes magnetic fields and radio waves to generate images of the internal tissues of the human body. Its greatest advantage lies in the absence of ionizing radiation, eliminating the risk of radiation-induced harm to the human body. Additionally, MRI exhibits high tissue contrast, enabling clear visualization of soft tissue structures, facilitating disease diagnosis and anatomical observations. Moreover, MRI can generate multi-planar images to provide comprehensive anatomical information. However, there are limitations due to the underlying imaging principles. Patients with implanted cardiac pacemakers, for example, are unable to undergo MRI scans, and the presence of other metallic implants may result in thermal injuries or adverse reactions. Furthermore, the high cost of MRI equipment and the need for specialized personnel for maintenance and operation restrict its widespread availability. Additionally, MRI scans are time-consuming, making long-duration procedures challenging to undertake. MRI was utilized to track porous iron-based MOF nanocarriers as integrated theranostic drug delivery systems (Böll et al., 2019).

US imaging employs the propagation and reflection characteristics of US waves to generate images of internal tissues in the human body. After being generated by an US transducer, US waves propagate through the tissue via a conductive medium, undergoing refraction, scattering, and absorption. Due to inconsistencies in the reflection of sound waves at different tissue structures and interfaces, images can be generated based on the reflected waves captured by the receiver (Boda-Heggemann et al., 2008). It allows high frame-rate, real-time imaging at relatively low cost without radiation, accurately capturing morphology and positional information of soft robots, making it well-suited for clinical applications. Recently, de Oliveira et al. (2022) proposed and validated a magnetically controlled bioinspired soft robot system based on US tracking and closed-loop control. Camera and US feedback enabled motion planning and control with small tracking errors. Wang et al. (2020) utilized US imaging to guide magnetic nanoparticles and tPA to blood clots and induce deformation for optimal thrombolysis, thus achieving thrombolytic therapy. Thus, US imaging is a versatile, accessible option for soft robot localization.

CT scanning offers rapid image acquisition, generating images within a few seconds. It is well-suited for emergency situations and urgent assessments, providing high resolution to visualize anatomical details and abnormalities. CT can generate multi-planar images, offering comprehensive anatomical information and is applicable to various body regions for diagnosing diseases and injuries. However, due to the use of X-rays in CT scanning, exposure to radiation increases the patient’s radiation dose. Consequently, frequent CT scans are not advisable, and it is contraindicated for pregnant women or patients with allergies to contrast agents. Shahriari et al. (2017) developed a CT-compatible remotely actuated needle-guiding robot that fuses CT and electromagnetic sensor data for needle tip localization and steering, enabling targeting of >5 mm lung nodules to reduce complications. While advantageous for anatomical delineation, judicious use of CT is warranted given radiation concerns.

## 5 Challenges and prospects

Soft robots, as an emerging robotic technology, hold vast promise for medical applications due to their flexible and compliant nature. Compared to conventional rigid robots, soft robots have gained preliminary usage in surgical assistance, targeted drug delivery, rehabilitation training and demonstrated great potential by virtue of their biomimetic properties. In order to optimize the clinical implementation of soft robots, they still face challenges related to the biocompatibility and degradability of materials, the extension of biomimetic approaches, and modeling.

The biocompatibility of soft robots is primarily influenced by the materials and components used in their construction. There are two common strategies in this regard. The first involves constructing the entire soft robot using materials that exhibit high biocompatibility. The second is creating an external “shell” that encapsulates materials and components with lower biocompatibility. For instance, this can be achieved by sputtering a layer of “biometal” titanium on the robot’s surface (Li N. et al., 2018), or by encapsulating the entire robot with hydrogels (Nasseri et al., 2023), PDMS (Niu et al., 2022), chitosan (Niu et al., 2017), etc. Furthermore, if the operational duration of the robot is brief and it can be degraded or expelled from the body afterwards, the requirements for biocompatibility can be moderately reduced. An example of this is the magnetically controlled capsule endoscope, which can be expelled from the body after completing its diagnostic function (Hua et al., 2022).

Most biological materials degrade over time, thus medical devices designed for temporary interventions should have degradable, integrative, or minimally disruptive clearance capabilities after completing specific tasks. Biodegradable robots that can be metabolized *in vivo* or *ex vivo* can serve as transient diagnostic and therapeutic tools to minimize harm to the body. Currently, aliphatic polyesters including poly(lactic-co-glycolic acid) (PLGA), poly(L-lactic acid) (PLLA), poly(D,L-lactic acid) (PDLLA), and poly(caprolactone) (PCL) are popular due to their biocompatibility and hydrolytic degradability. Furthermore, introducing bio-cleavable bonds into hydrogel structures enables degradable soft robotics using composite matrix precursor polymerization or helical microstructures from hydrogels. PEGDA and GelMA can serve as hydrogel precursors. Additionally, naturally-derived alginate and chitosan are useful for degradable robots. Compared to conventional approaches, biodegradable robots can enable cheaper, safer, and more efficient surgeries (Llacer-Wintle et al., 2021). Goudu et al. (2020) developed fully degradable soft millimetric robots with encoded 3D magnetic anisotropy for static and dynamic shape control. As a proof of concept, reversible deformations were demonstrated in hydrogel millimetric grippers in glycerol and biologically-relevant media. The grippers executed cargo grabbing, rolling transport, and release through magnetic field modulation. Complete degradation of the grippers by MMP-2 was achieved at physiologically-relevant concentrations. Additionally, biocompatibility testing of the degradation products using human umbilical vein endothelial cells showed no acute toxicity.

As natural systems have evolved over billions of years, bio-inspired designs can be leveraged to enhance soft robotics. By mimicking the structures, morphologies, functions, and processes of biological organisms, the stability, adaptability, and capabilities of soft robots can be improved. Through biomimetic designs of structure and form, soft robots can achieve increased robustness and adaptability to complex environments. Functional biomimesis, such as replicating locomotion, sensing, and control schemes from nature, can enable more efficient energy conversion, flexible movements, and intelligent control. By studying biological self-assembly and self-healing, soft robots can possess desirable resilience and self-repairing abilities. The applications of bionics in soft robotics extend beyond morphological designs to algorithmic control as well, for example, through ant colony optimization, neural networks, and other bio-inspired control schemes to augment the autonomous mobility and adaptability of soft robots (Wang and Chortos, 2022).

When soft robots are utilized for biomedical applications, accurate modeling is highly desired to minimize adverse impacts on the human body. However, unlike traditional robots made of rigid components, soft robots employ materials that often exhibit nonlinear characteristics such as viscoelasticity, large strains, or deformations, making kinematic and dynamic modeling extremely challenging. Moreover, soft robots possess near infinite degrees of freedom, necessitating novel control approaches tailored for soft robots. Currently, finite element analysis (FEA) can provide relatively precise models. To further enhance control accuracy of soft robot motion, future work may integrate deep learning artificial intelligence (AI) to optimize modeling and control from large datasets, while developing more sophisticated sensors for feedback of soft robot states to enable more precise closed-loop control. In summary, precise modeling and control of soft robot dynamics and interactions remain open challenges vital for advancing biomedical applications of soft robotics. Advanced numerical methods, data-driven modeling, AI-enabled control algorithms, and real-time state feedback through innovative sensory solutions will likely play key roles in realizing the immense promise of soft robots to safely augment human capabilities and improve wellbeing.

## 6 Conclusion

This review has provided a comprehensive overview of recent advancements in soft robotics for biomedical applications. Significant progress has been made in in vitro and *in vivo* contexts owing to the unique capabilities of soft robots including flexibility, biocompatibility, adaptability, and miniaturization. For *in vitro* applications, soft robots show potential for cell culture engineering, surgical assistance, drug screening, and wearable assistive devices. *In vivo*, they enable minimally invasive diagnosis, targeted drug delivery, biopsy sampling, and catheter interventions by safely navigating the body.

Several key aspects were highlighted that are propelling soft robot development. Intelligent stimuli-responsive materials and bioinspired designs are enhancing functionality while ensuring biocompatibility. Rapid manufacturing techniques like 3D printing facilitate iterative prototyping and customization. Untethered propulsion methods utilizing chemical fuels, biological motility, or external wireless actuation overcome previous constraints, expanding the range of accessible sites. Precision navigation and localization are enabled by tracking modalities like FI and US imaging. Algorithmic control further augments soft robotic capabilities.

While still an emerging field, soft robotics holds immense clinical promise. With continuing advances in materials, manufacturing, propulsion, and localization, soft robots are poised to revolutionize minimally invasive diagnosis and therapies. Areas warranting further research include biocompatibility, biomimetic designs, degradability, and on-board power supplies. Seamless integration with medical imaging for localization and control also remains an open challenge. As these capabilities mature, soft robotics could enable the next-generation of intelligent diagnostic and therapeutic technologies to improve patient outcomes.
